# Evaluation of multiple freeze–thaw cycles on growth factor and cytokine stability in canine platelet lysate: PDGF, TGF-*β*, and TNF-*α* preservation across cycles

**DOI:** 10.3389/fvets.2026.1817599

**Published:** 2026-04-29

**Authors:** Scarlett M. Sumner, Maria C. Naskou

**Affiliations:** 1 Department of Clinical Sciences, College of Veterinary Medicine, Auburn University, Auburn, AL, United States; 2 Department of Pathobiology, College of Veterinary Medicine, Auburn University, Auburn, AL, United States; 3 Scott-Ritchey Research Center, College of Veterinary Medicine, Auburn University, Auburn, AL, United States

**Keywords:** canine, growth factor, lyophilized, platelet lysate, platelet rich plasma

## Abstract

**Introduction:**

Platelet-rich plasma (PRP) and platelet lysate (PL) are biologically active products used in regenerative medicine because of their high concentrations of growth factors, cytokines, and other bioactive molecules that promote tissue repair and modulate inflammation. Freeze–thaw cycles are commonly used to activate PRP and induce growth factor release. While the optimal number of freeze–thaw cycles has been evaluated in human PRP, this parameter has not been investigated in canine PRP products. The objective of this study was to evaluate platelet counts and concentrations of platelet-derived growth factor (PDGF), transforming growth factor–beta (TGF-*β*), and tumor necrosis factor–alpha (TNF-*α*) in a commercially available, pooled, freeze-dried canine PRP product subjected to multiple freeze–thaw cycles (1, 3, 4, 5, and 10). We hypothesized that (1) a significant proportion of platelets would be lysed by the fourth freeze–thaw cycle and (2) growth factor concentrations would decrease, whereas TNF-*α* concentrations would increase by the 10th freeze–thaw cycle.

**Methods:**

Six vials of a commercially available canine PRP product from three separate manufacturing lots were evaluated. Each vial was reconstituted according to the manufacturer’s instructions and stored at −80 °C for 12–24 h. Samples were thawed in a dry bath at 37 °C. Additional freeze–thaw cycles were performed using liquid nitrogen, with thawing at 37 °C for samples undergoing 3, 4, 5, and 10 cycles. A complete blood count was performed before freezing and after each freeze–thaw cycle. Concentrations of PDGF, TGF-*β*, and TNF-*α* were quantified using enzyme-linked immunosorbent assays (ELISAs).

**Results:**

Initial platelet and white blood cell counts were consistent with manufacturer-reported values. Platelet counts were significantly reduced following 4, 5, and 10 freeze–thaw cycles compared with initial PRP values. Concentrations of PDGF, TGF-*β*, and TNF-*α* were largely conserved across freeze–thaw cycles, although significant inter-sample variability was observed.

**Discussion:**

Four to five freeze–thaw cycles effectively lysed platelets while preserving PDGF and TGF-*β* concentrations for at least 10 cycles. However, significant variability in growth factor and cytokine content was evident, even in a commercially available product. Further investigation is warranted to better define the effects of freeze–thaw cycles on activating PRP intended for clinical therapeutic use.

## Introduction

Platelet-rich plasma (PRP) and platelet lysate (PL) are biologically active products used in regenerative medicine due to their high concentrations of growth factors, cytokines, and bioactive molecules that promote tissue repair and modulate inflammation (1–4). PRP is typically prepared from whole blood by centrifugation, resulting in a concentrated platelet fraction enriched with their associated growth factors such as platelet-derived growth factor (PDGF), transforming growth factor-beta (TGF-*β*), vascular endothelial growth factor (VEGF), and hepatocyte growth factor (HGF) (4–6). Upon activation or lysis, platelets release these factors, which play critical roles in angiogenesis, fibroblast proliferation, and extracellular matrix remodeling (7–9).

PL, produced by repeated freeze–thaw cycles or chemical activation of a platelet concentrate such as PRP, is increasingly used in therapeutic applications and as a replacement for fetal bovine serum in cell culture (1, 10–12). Lysate preparation aims to maximize growth factor release while maintaining stability, but there is concern that repeated freeze–thaw cycles can degrade proteins and reduce bioactivity. Strandberg et al. found that optimal human PL was obtained with 3–5 freeze–thaw cycles, with some growth factors declining with further cycles (13). McClain and McCarrel reported growth factor and enzyme concentrations in equine PRP following calcium activation, a single snap freeze, and four storage methods across three time points. A single snap freeze led to an increase in matrix metalloproteinase-9 (MMP), no change in insulin-like growth factor (IGF-1) and platelet-derived growth factor (PDGF-BB), and a small decrease in transforming growth factor (TGF-*β*1) compared to PRP activated with calcium. Additional freeze–thaw cycles were not evaluated, but the storage of equine PRP for clinical applications was determined acceptable at −80 °C for 1 month or in liquid nitrogen for 6 months to maintain PDGF-BB and TGF-*β*1 concentrations, but IGF-1 concentrations were expected to decrease (14).

Canine PL has been studied as a potential therapeutic in regenerative medicine (15, 16), including a case report describing *in vivo* use in a dog with flexor carpi ulnaris tendinopathy (17), as well as investigations evaluating its antimicrobial effects *in vitro* (18, 19). The most commonly reported method for platelet lysis is freeze–thaw cycling. Growth factors and cytokines, including PDGF-BB, TGF-*β*1, VEGF, hepatocyte growth factor (HGF), and tumor necrosis factor-alpha (TNF-*α*), have been evaluated in multiple formulations of canine PL, all of which were activated using five freeze–thaw cycles (5). Growth factor release (PDGF-BB, TGF-*β*1, VEGF, and TNF-*α*) under different activation protocols for canine PRP, including a single freeze–thaw cycle, has been analyzed, and the findings indicate that a single freeze–thaw cycle produces a moderate release of growth factors comparable to calcium chloride activation, although less robust than activation with human thrombin. The data also support that platelet activation is more influential on growth factor concentration than platelet concentration (20). However, the ideal number of freeze–thaw cycles and their effect on growth factors specific to canine platelet lysate have yet to be evaluated.

This study aimed to evaluate the platelet counts and concentration of PDGF, TGF-*β*, and TNF-*α* in a commercially available, pooled, freeze-dried canine PRP product across multiple freeze–thaw cycles (1, 3–5, 10). We hypothesized that (1) a significant number of platelets would be lysed by the fourth freeze–thaw cycle and (2) growth factor concentrations would decrease by the tenth freeze–thaw cycle.

## Methods

### PRP preparation

Six vials of a commercially available PRP (VetStem PrecisePRP Canine, Poway, CA, USA) from three separate lots were used in this study. Each vial was reconstituted according to the manufacturer’s instructions. Briefly, 8 mL of sterile water was added to each vial using aseptic technique. A swirling motion of the vial was performed to achieve homogeneous rehydration of the PRP. Each vial was aliquoted into 0.5 mL Eppendorf tubes and stored at −80 °C for 12–24 h. A complete blood count (CBC) was performed prior to freezing.

### PRP freeze–thaw

Each sample was thawed in a dry bath at 37 °C. This constituted samples with one freeze–thaw cycle. Additional freeze–thaw cycles were performed using liquid nitrogen and a dry bath at 37 °C for samples undergoing 3, 4, 5, and 10 freeze–thaw cycles. Additional CBCs were performed following each freeze–thaw cycle.

### Growth factor and cytokine quantification

Growth factors and cytokines were quantified using enzyme-linked immunosorbent assays (ELISAs) validated for canine plasma: platelet-derived growth factor (PDGF-BB) (Canine PDGF-BB ELISA Kit, Invitrogen, ThermoFisher Scientific, CA, USA), transforming growth factor-beta 1 (TGF-*β*1) (Human, Mouse/Rat/Porcine/Canine TGF-*β*1 Quantikine ELISA Kit, R&D Systems, MN, USA), and tumor necrosis factor-*α* (TNF-*α*) (Canine TNF-alpha (TNF) ELISA kit, Invitrogen, ThermoFisher Scientific, CA, USA). ELISAs were performed in duplicate, and absorbance was read on a BioTek Synergy H1 multimode reader (Agilent, CA, USA) at the specified wavelength absorption and corrected according to the manufacturer’s instructions.

### Statistical analysis

All data were analyzed using a statistical analysis program (GraphPad Prism; GraphPad Software Inc., San Diego, CA, USA). For statistical analysis of platelet and white blood cell counts, a repeated-measures ANOVA was performed. Assumptions of normality were assessed using the Shapiro–Wilk test at each time point. *Post-hoc* pairwise comparisons between time points were performed using paired t-tests with a Bonferroni correction to control for multiple comparisons.

For growth factors and cytokine comparisons, repeated-measures ANOVA was used to assess the effects of freeze–thaw cycles and individual samples. Assumptions were tested using Shapiro–Wilk and Levene’s tests. Tukey’s honestly significant difference test was applied for *post-hoc* comparisons. Statistical significance was set at a *p*-value of < 0.05.

## Results

### Platelet and white blood cell count

**Figure 1:**
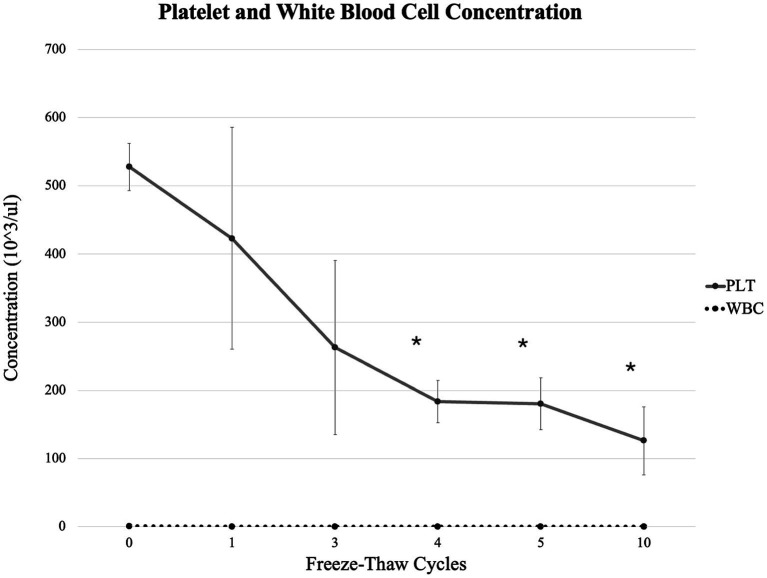
Platelet and white blood cell count in PRP (mean ± standard deviation) following 1, 3, 4, 5, and 10 freeze–thaw cycles. Asterisk signifies a significant difference from PRP (0 freeze–thaw cycles). WBCs, white blood cells; PLT, platelets; PRP, platelet-rich plasma.

Platelet and white blood cell counts are presented in Figure 1. White blood cell counts were minimal (<450/μL). The PRP platelet count was 527.3 ± 34.5 × 10^3^/μL (mean ± standard deviation). Platelet counts were significantly different between PRP and after 4, 5, and 10 freeze–thaw cycles (*p* = 0.000066, 0.000217, and 0.000024, respectively).

### Growth factor and cytokine concentrations

**Figure 2:**
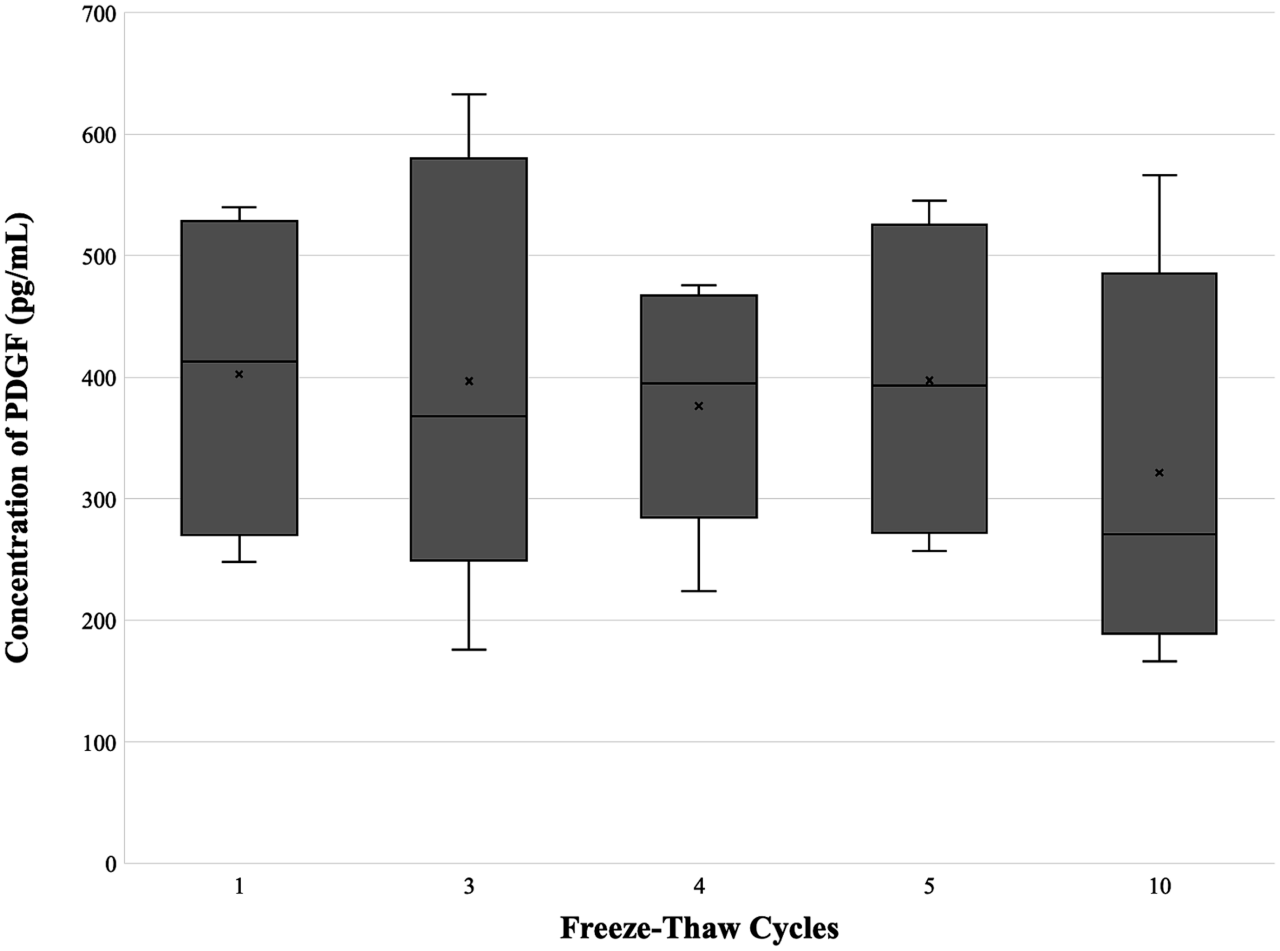
Concentration of PDGF (*n* = 6) following 1, 3, 4, 5, and 10 freeze–thaw cycles. A significant difference was not identified within the various freeze–thaw cycles. Data represented as mean ± SD. PDGF, platelet-derived growth factor.

**Figure 3:**
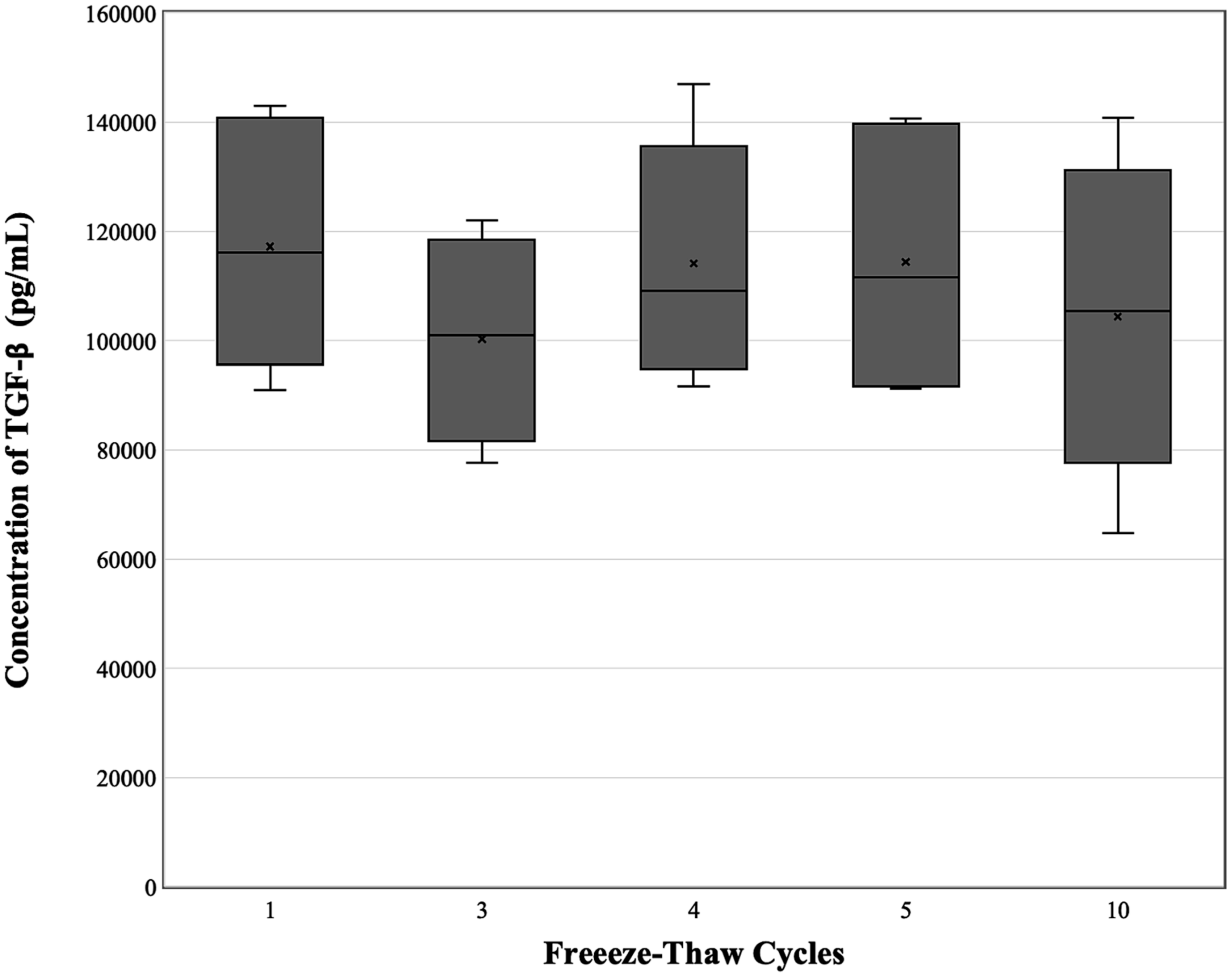
Concentration of TGF-*β* (*n* = 6) following 1, 3–5, and 10 freeze–thaw cycles. A significant difference was not identified within the various freeze–thaw cycles. TGF-*β*, transforming growth factor-beta.

**Figure 4:**
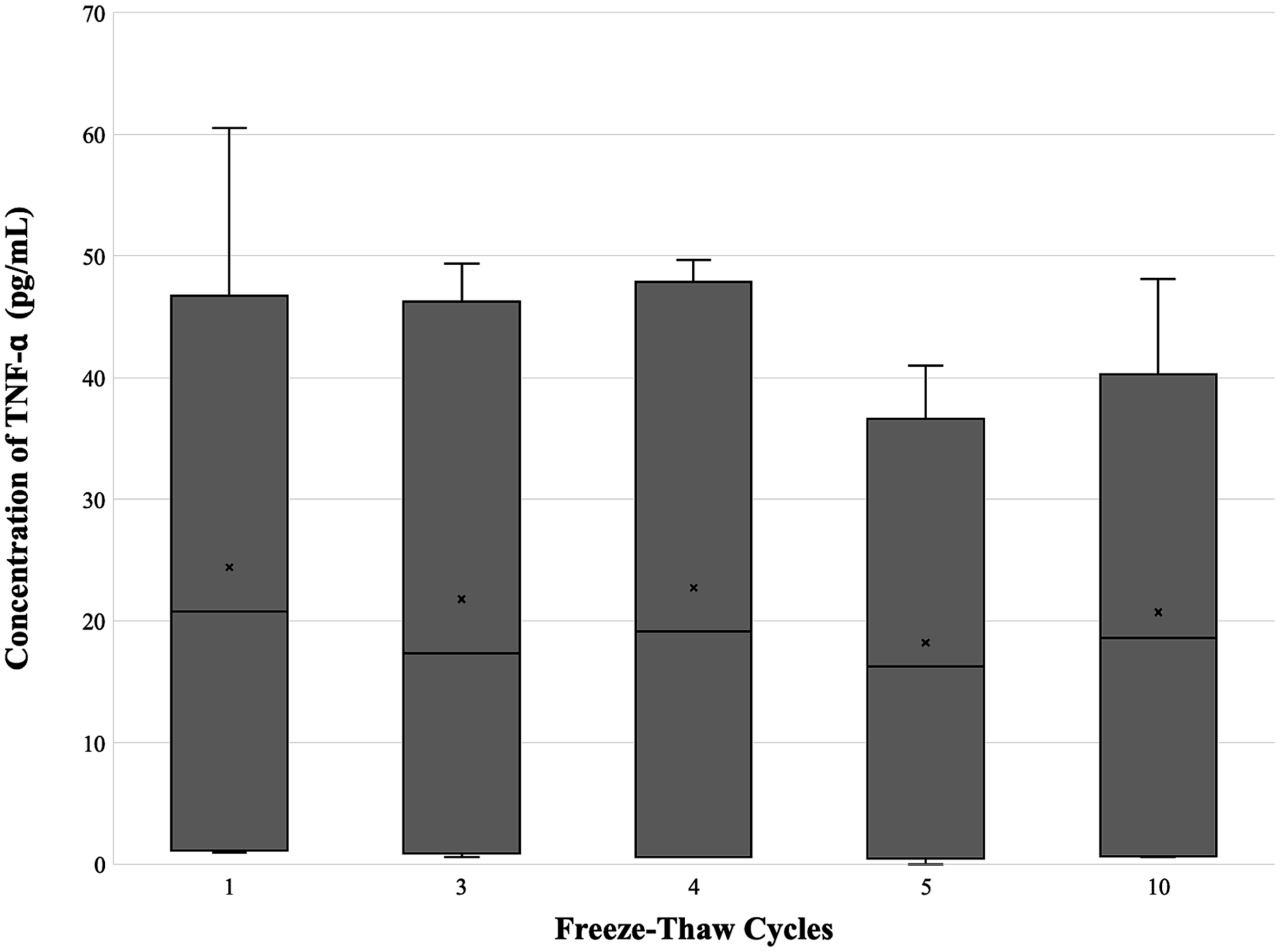
Concentration of TNF-*α* (*n* = 6) following 1, 3, 4, 5, and 10 freeze–thaw cycles. A significant difference was not identified within the various freeze–thaw cycles. TNF-*α*, tumor necrosis factor-alpha.

Growth factor concentrations are depicted in Table 1 and Figures 2–4. For PDGF (Figure 2) and TNF-*α* (Figure 4), the freeze–thaw effect was not significant (*p* = 0.283, *p* = 0.401), while the differences between PL samples were highly significant (*p* < 0.001, *p* < 0.001). For TGF-*β* (Figure 3), the freeze–thaw effect was significant (*p* = 0.007), and the differences between PL samples were also highly significant (*p* < 0.001). However, the Tukey HSD *post-hoc* comparisons for freeze–thaw cycles did not identify significant pairwise differences for TGF-*β*. Comparing PL lots (*n* = 3), no differences were noted between freeze–thaw cycles for PDGF (*p* = 0.525), TGF-*β* (*p* = 0.2636), or TNF-*α* (*p* = 0.3725). A significant difference between lots was noted for each factor (PDGF, *p* = 0.0003; TGF-*β*, *p* = <0.000; TNF-*α*, *p* = <0.0001). A trend was noted for PDGF concentration to drop after the fourth or fifth freeze–thaw cycle and for TGF-*β* to drop after the fifth freeze–thaw cycle.

**Table 1:** Concentrations of PDGF, TGF-*β*, and TNF-*α* across freeze–thaw cycles.

Growth factor/cytokine	Lot	Sample	Freeze–thaw cycle				
			1	3	4	5	10
PDGF pg./mL	1	1	524.18	562.16	464.38	488.86	566.26
	2	2	511.42	632.74	380.82	518.86	457.76
		3	539.86	440.62	475.40	545.16	270.32
	3	4	314.42	273.48	408.78	277.16	270.76
		5	277.28	295.50	305.06	256.86	166.26
		6	248.04	175.72	223.72	297.30	196.72
TGF-*β* pg./mL	1	1	127,100.55	117,148.65	146,921.25	140,741.7	140,875.05
	2	2	142,949.70	122,043.75	131,905.95	139,282.65	128,021.70
		3	140,018.25	112,053.00	118,453.20	113,690.55	114,985.65
	3	4	104,960.25	89,806.35	95,897.40	109,499.55	95,808.15
		5	97,191.75	82,969.50	99,716.10	91,214.55	64,775.85
		6	90,986.25	77,640.60	91,626.30	91,884.30	81,878.70
TNF-*α* pg./mL	1	1	60.51	45.18	49.66	35.12	35.56
	2	2	42.14	49.36	47.24	40.98	37.71
		3	39.54	33.44	35.83	30.21	48.09
	3	4	2.01	0.97	0.59	0.00	0.67
		5	1.17	1.18	2.47	2.31	1.63
		6	0.91	0.60	0.55	0.61	0.56

PDGF, platelet-derived growth factor; TGF-*β*, Transforming growth factor-beta; TNF-*α*, tumor necrosis factor-alpha.

## Discussion

Platelet counts of the reconstituted PRP (527.3 ± 34.5 × 10^3^/μL, mean ± standard deviation) were consistent with the manufacturer’s reported concentration of 4 billion platelets per vial, corresponding to approximately 500,000 platelets/μL (21). White blood cell counts were minimal (<450/μL) and consistent with the manufacturer’s report of less than 1,500/μL (21). Platelet counts decreased with each additional freeze–thaw cycle, with significant reductions from baseline noted after 4, 5, and 10 freeze–thaw cycles, confirming our first hypothesis.

Growth factors, PDGF and TGF-*β*, were overall conserved across freeze–thaw cycles, leading to the rejection of our second hypothesis. Strandberg et al. evaluated the effect of freeze–thaw cycles on human platelet concentrate obtained via platelet pheresis with a starting thrombocyte particle concentration (platelet count) of 1.6 × 10^9^/μL, which was far more concentrated than the PRP utilized in this study. PDGF in human PL was noted to significantly increase with additional freeze–thaw cycles, reaching 184.5 ± 21.9 ng/mL (184,500 pg./mL) at cycle 30. PDGF levels were lower in canine PL in this study, with mean values ranging from 321 to 402 pg./mL across cycles and showed a trend toward a decrease after 10 freeze–thaw cycles. Additional freeze–thaw cycles may have identified further significant changes. PDGF levels in this study were also lower across cycles than previously reported levels of PDGF in canine PL from fresh PRP standardized to 0.8–1 × 10^6^/μL and subjected to five freeze–thaw cycles (1,988.00 ± 1,792.94 pg./mL), and this difference may have been due to the difference in starting platelet concentration (5). Weak-to-moderate correlation has been observed between PRP platelet concentration and growth factor concentrations. Additionally, PDGF levels have been noted to vary widely between PRP production techniques and activation methods, with freeze–thaw providing moderate activation (20). In this study, the PRP was prepared from freeze-dried platelets, and previous studies have confirmed the preservation and even increased levels of PDGF in freeze-dried platelet products from mice, horses, and dogs compared to fresh PRP (22–24).

Strandberg et al. noted a peak in TGF-*β* levels at 3 and 5 freeze–thaw cycles and a decline in levels between 10 and 30 freeze–thaw cycles without a significant difference between 5 and 10 cycles (13). A trend toward decreased TGF-*β* was observed in some samples after 10 freeze–thaw cycles, and further declines might have been detected with a larger sample size or additional freeze–thaw cycles. Our data might signify a similar trend if additional freeze–thaw cycles were performed. However, a significant increase in TGF-*β* was not noted between 1 and 3 freeze–thaw cycles, as reported by Stranberg et al. Rather, there was a trend for TGF-*β* to decrease at three freeze–thaw cycles with a rebound following four freeze–thaw cycles. The reason for this trend is unknown, but it was consistent across individual samples (Figure 4). The overall average TGF-*β* levels in canine PL across freeze–thaw cycles (100,276–117,201 pg./mL) were similar to and higher than the average at the optimal 3–5 freeze–thaw cycles for human PL (94,000 ± 2,900 pg./mL and 96,800 ± 1,900 pg./mL), which is interesting given the difference in platelet concentration between the two starting products (13). In contrast to the PDGF levels, the TGF-*β* levels in this study were also similar to those previously reported in our laboratory on canine PL produced from fresh PRP standardized to 0.8–1 × 10^6^/μL and subjected to five freeze–thaw cycles (5).

TNF-*α*, a pro-inflammatory cytokine, remained within the range previously reported for young (1–4 years), healthy dogs with no significant changes across freeze–thaw cycles (25). Levels observed in this study were higher than those previously reported in canine PL produced via the manual centrifugation of whole blood, and this may be a variation of the starting PRP manufacturing (5). TNF-*α* levels were lower than previously reported in equine PL, which was also manufactured from the manual centrifugation of whole blood (26). The product in this study was produced using apheresis rather than the manual centrifugation of collected whole blood. This manufacturing difference, along with the freeze–drying process and potential donor variation, including donor age and platelet count, may also play a role in cytokine expression. Pirrone et al. demonstrated age-associated differences in serum TNF-*α* concentrations, with higher median values observed in senior dogs (>11 years; 43 pg./mL) compared with young dogs (1–4 years; 14.40 pg./mL), accompanied by inter-individual variability within each age group (interquartile range 2.10–54.17 pg./mL and 9.06–112.37 pg./mL, respectively) (25). Both equine and canine studies, along with this study, noted significant differences between individual samples.

As a proinflammatory cytokine, TNF-*α* has been associated with cell death, metabolic derangements, impaired glucose tolerance, tick-borne diseases, and sepsis (27–30). In human medicine, TNF-*α* inhibitors are used to treat inflammatory autoimmune diseases such as rheumatoid arthritis and ulcerative colitis. However, a side effect of the TNF-*α* inhibitors is an increased risk of infections, and they have been noted to worsen the prognosis for patients with abscesses (29, 30). Additionally, exposing (or priming) mesenchymal stem cells to inflammatory cytokines, such as TNF-*α* and interferon (IFN)-*γ*, before using the stem cells or their extracellular vesicles as a treatment enhanced their anti-inflammatory effects, immunomodulating potential, and their therapeutic effects in treating lung injury and inflammatory bowel disease (31–33). It is worth noting that the concentration of TNF-*α* used for priming, 20 ng/mL (20,000 pg./mL), was much higher than the PL concentration in this study. Additional studies are needed to determine the ideal concentration of TNF-*α* and its biological effects on PL as a therapeutic, taking into consideration that the ideal concentrations may differ based on the desired use of the final product, and freeze–thaw cycles would have limited effect on the concentration.

Individual variability is a common obstacle encountered with biologic products, with differences noted between biological replicates, technical replicates, and manufacturing techniques (34–38). The ability to pool PL can help reduce individual variability, and pooled equine products have shown advantages in reducing TNF-*α* from monocytes in cell culture (39), as well as proliferation and collagen gene expression from synovial cells (40). Interestingly, even though the commercial product evaluated in this study is a pooled product, incorporating up to 32 donors (21), significant variability between PRP lots was still noted for PDGF, TGF-*β*, and TNF-*α*. As previously mentioned, the platelet count and white blood cell count consistently matched the manufacturer’s standard. Given these consistencies and the pooling from many individuals, growth factor and cytokine variability may have been due to platelet contents, platelet function, or variability in platelet activation.

The freeze–thaw technique using liquid nitrogen and a dry bath was chosen based on established laboratory protocols within the authors’ laboratory, which have been evaluated in our previous study using five freeze–thaw cycles, and has the benefit of processing multiple freeze–thaw cycles (5, 19). Protocols for freeze–thaw activation of PL vary. Strandberg et al. evaluated human PL after up to 30 freeze–thaw cycles using −70 °C for 10 min followed by a warm water bath at 37 °C for 7.5 min (13). A systematic review by Palombella et al. noted most freeze–thaw protocols reported 1–5 cycles with a − 80 °C freeze and a 37 °C thaw for the activation of PL. Other reported temperatures included −30 °C and −40 °C for the freeze and 4 °C for the thaw (12). Liquid nitrogen may not be available to many veterinary practitioners, and for commercial products, the freeze–thaw may need to be incorporated during the product development. Commercially available pooled human PLs for use in cell culture recommend aliquoting the product and storing at −20 to −80 °C, with some preferring the −80 °C and then thawing for use (Human Platelet Lysate, Sigma Aldrich, St. Louis, MO, USA; pooled human platelet lysate, ELK Bioscience, Doylestown, PA, USA; PharmaOnco, Human Platelet Lysate (HPL), Creative Biolabs, Shirley, NY, USA). Further research is needed to confirm the stability of PL at −20 °C, which would enhance its usability for more veterinary practitioners.

Limitations of this study include a limited sample size and an uneven number of samples from each lot. Due to the uneven numbers of vials available per lot, the investigators evaluated differences between lots and between vials. Additionally, a limited number of factors were tested. PDGF-BB, TGF-*β*1, and TNF-*α* were tested due to the availability of ELISAs validated for canine samples and for comparison across multiple studies available in the literature. However, these studies do not cover all diverse growth factors and cytokines present in PL. Additional growth factors such as HGF, IGF-1, and VEGF are present in canine platelet products and may be affected by the freeze–thaw process. Strandberg et al. noted no difference in VEGF levels across up to 30 freeze–thaw cycles in human PL, and this has not yet been confirmed in canine PL (13). Moreover, growth factor concentration represents only one aspect of understanding the effect of freeze–thaw cycles on PL as a therapeutic. A study that included a commercial pooled human PL (AlloPL, Germany) noted that the commercial product had some of the highest growth factor contents, including TGF-*β*1 and PDGF-AB, compared to other platelet concentrates and had downregulated COX1 expression in tenocytes *in vitro*, suggesting the potential for pain relief and anti-inflammatory benefits. However, an increase in tenocyte viability and gene expression of extracellular matrix proteins was not observed despite the higher growth factor content (41). The effect of the freeze–thaw cycles was not evaluated in cell function or *in vivo*, and it is important to note that the full release of growth factors may not be ideal for all clinical applications of PL. Exogenous activation may be helpful in determining growth factor potential when comparing PRP or PL formulations. It may also be beneficial for topical treatments with daily applications or when specific growth factor concentrations are desired to be formulated into hydrogels or other specialized formulations for controlled release. Endogenous activation can also occur with exposure to components such as collagen (42, 43), which may occur with the application of PL to a site of injury. Additionally, allowing endogenous activation may result in a more sustained release of growth factors at the treatment site over prolonged periods of time (43). Freeze–thaw cycles may also affect platelet function and reduce the potential for continued synthesis of growth factors (44, 45). However, a study evaluating lyophilized canine PRP, which was frozen once at −80 °C during its preparation, had similar or increased levels of TGF-*β*1, PDGF, and VEGF compared to fresh PRP, and had a maximum release of growth factors at 24 h *in vitro*. Moreover, continued release was still noted at 72 h. Dosing was also emphasized in this study, with lower doses (5% vs. 20% total volume) of both fresh and lyophilized PRP benefiting wound closure on an *in vitro* fibroblast scratch test (24). These data highlight the need for continued investigation into platelet-derived therapeutics for the best product formulations and applications. Additional studies are needed, both *in vitro* and *in vivo*, to better understand the effects of platelet activation on the biological and therapeutic activity of PL.

In conclusion, platelets were significantly lysed from PRP following 4, 5, and 10 freeze–thaw cycles. No significant difference was noted for PDGF, TGF-*β*, and TNF-*α* across the cycles. Four to five freeze–thaw cycles are a reasonable range for producing PL from platelet concentrates, while higher numbers of cycles may lead to reductions in PDGF and TGF-*β*. Further investigation is needed to determine the effects of freeze–thaw cycles on the therapeutic potential of PRP.

## Data Availability

The original contributions presented in the study are included in the article/supplementary material, further inquiries can be directed to the corresponding author.
